# Epigenome-wide analysis of common warts reveals aberrant promoter methylation

**DOI:** 10.7150/ijms.39261

**Published:** 2020-01-14

**Authors:** Laith N. AL-Eitan, Mansour A. Alghamdi, Amneh H. Tarkhan, Firas A. Al-Qarqaz

**Affiliations:** 1 Department of Applied Biological Sciences, Jordan University of Science and Technology, Irbid 22110, Jordan; 2 Department of Biotechnology and Genetic Engineering, Jordan University of Science and Technology, Irbid 22110, Jordan; 3 Department of Anatomy, College of Medicine, King Khalid University, Abha 61421, Saudi Arabia; 4 Department of Internal Medicine, Jordan University of Science and Technology, Irbid 22110, Jordan; 5 Division of Dermatology, Department of Internal Medicine, King Abdullah University Hospital, Jordan University of Science and Technology, Irbid 22110, Jordan

**Keywords:** wart, HPV, methylation, promoter, epigenetics

## Abstract

Epigenetic alteration of host DNA is a common occurrence in both low- and high-risk human papillomavirus (HPV) infection. Although changes in promoter methylation have been widely studied in HPV-associated cancers, they have not been the subject of much investigation in HPV-induced warts, which are a temporary manifestation of HPV infection. The present study sought to examine the differences in promoter methylation between warts and normal skin. To achieve this, DNA was extracted from 24 paired wart and normal skin samples and inputted into the Infinium MethylationEPIC BeadChip microarray. Differential methylation analysis revealed a clear pattern of hyper- and hypomethylation in warts compared to normal skin, and the most differentially methylated promoters were found within the *EIF3EP2*, *CYSLTR1*, *C10orf99*, *KRT6B*, *LAMA4*, and *H3F3B* genes as well as the *C9orf30* pseudogene. Moreover, pathway analysis showed that the *H3F3A*, *CDKN1A*, and *MAPK13* genes were the most common regulators among the most differentially methylated promoters. Since the tissue samples were excised from active warts, however, this differential methylation could either be a cellular response to HPV infection or an HPV-driven process to establish the wart and/or promote disease progression. Conclusively, it is apparent that HPV infection alters the methylation status of certain genes to possibly initiate the formation of a wart and maintain its presence.

## Introduction

Epigenetics is the study of heritable changes in gene expression that are not caused by changes to the DNA sequence itself, but by covalent modifications such as DNA methylation (DNA-M) 1. Mammalian DNA-M, which primarily involves the addition of a methyl group to a cytosine base in a CpG dinucleotide, results in increased gene expression when it occurs at higher levels within the gene's body instead of its promoter region 2. On a similar note, promoter methylation is of particular epigenetic importance because the vast majority of those located upstream of a gene contain a CpG island, the latter of which is a region with a high concentration of CpG sites 3. In contrast to the hypermethylated CpG sites scattered throughout the human genome, CpG islands are not methylated, and the methylation of CpG islands initiates remodeling mechanisms that ultimately result in gene silencing 4, 5.

The methylation status of promoters is integral to maintaining normal expression levels of the genes they regulate. In fact, promoter hypermethylation is a key part of cancer development and progression, as it results in the silencing of tumor suppressor gene expression 6. In addition, host promoter hypermethylation has also been implicated in infections by both oncogenic and non-oncogenic viruses such as the human papillomaviruses (HPV) 7. HPV comprises a family of double-stranded DNA viruses that exclusively infect the basal epithelium of the skin and mucosa 8. The majority of HPV infections are asymptomatic and resolve without the need for medical intervention but, depending on the individual and the HPV type, can also result in a number of malignancies and dermatological diseases 9. One such condition is the wart, which arises due to the benign proliferation of HPV-infected epithelial keratinocytes 10. The most prevalent type of wart is the common wart, which accounts for nearly 70% of all cutaneous warts encountered in clinical settings 11. As a result of their benign nature, common warts are subject to a much lesser degree of scrutiny than other HPV-associated diseases.

The impermanent nature of cutaneous warts strongly suggests that epigenetic changes are involved in the mechanism of wart formation and their eventual disappearance. However, a paucity of information exists regarding the methylation status of cutaneous warts, especially in the context of the promotor regions. Therefore, the primary objective of the current study was to provide an exploratory survey of the genome-wide changes in promoter methylation patterns in cutaneous warts compared to healthy skin.

## Materials and Methods

### Study participants

Ethical approval to conduct this study was obtained from Jordan University of Science and Technology's (JUST) Institutional Review Board (IRB). Twelve Arab males presenting with common warts were recruited from the general population after providing written informed consent. Shave biopsies of common warts and adjacent normal skin were performed, allowing paired tissue samples (wart and normal skin) to be obtained from each participant.

### Whole genome bisulfite sequencing

A QIAamp DNA Mini Kit (Qiagen, Germany) was used to perform DNA extraction, and optional RNase A digestion was incorporated. DNA purity and integrity were determined by means of the BioTek PowerWave XS2 Spectrophotometer (BioTek Instruments, Inc., USA) and agarose gel electrophoresis, respectively. Genomic DNA that fulfilled our standards for quality and quantity were shipped on dry ice to the Australian Genome Research Facility (AGRF) in Melbourne, where the quality was further ascertained by the QuantiFluor® dsDNA System (Promega, USA). The Zymo EZ DNA Methylation Kit (Zymo Research, USA) was utilized in order to perform bisulfite conversion on the 24 samples. Lastly, the samples were inputted into the Infinium MethylationEPIC BeadChip microarray (Illumina, USA) for a genome-wide interrogation of over 850,000 CpG sites.

### Data processing

RnBeads, a computational R package, was adapted to process and analyze the raw intensity data (IDAT files) from the BeadChip 12. Quality control, preprocessing, batch effects adjustment, and normalization were carried out on all probes and samples according to the RnBeads package pipeline.

### Differential methylation and statistical analysis

**Figure 1:**
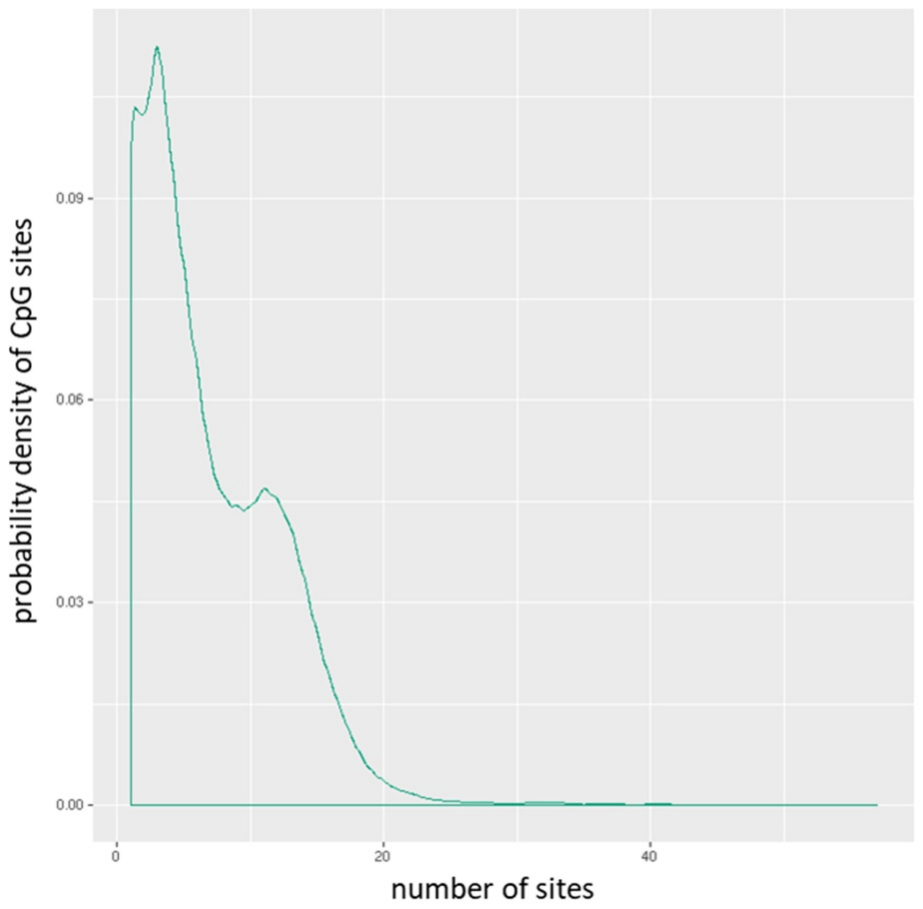
Distribution of CpG sites per promoter.

**Figure 2:**
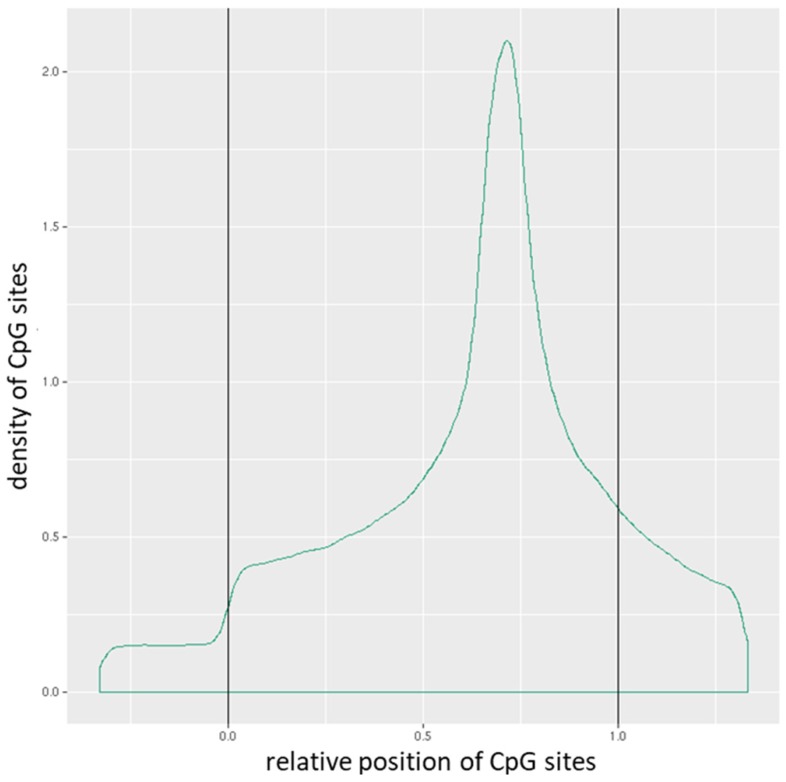
Distribution of CpG sites across promoters. The relative coordinates of 0 and 1 correspond to the start and end coordinates of promoters. Coordinates smaller than 0 and greater than 1 denote flanking regions normalized by region length.

The mean of the mean β (mean.mean β) values of all the interrogated CpG sites in each promoter were computed. The distribution of CpG sites per promoter is shown in **Figure 1**, while **Figure 2** depicts the distribution of CpG sites across promoters. DM for each promoter was calculated using the following three measures: the mean.mean β difference between warts (W) and normal skin (NS), the log_2_ of the mean quotient in β means across all CpG sites in a promoter, and the adjusted combined p-value of all CpG sites in the promoter using a limma statistical test 12, 13. Furthermore, these three measures were used to create a combined ranking, in which promoters that exhibit more DM are assigned a lower combined rank 12. Promoters were sorted from smallest to largest using the combined rank score, and the top-ranking 1000 DM promoters were selected for further analysis. In order to correct for multiple testing, the Benjamini-Hochberg procedure was utilized to set the false discovery rate (FDR) at 5%.

### Gene ontology enrichment analysis

Enrichment analysis for gene ontology (GO) terms associated with the top-ranking 500 DM promoters was performed using the GO consortium 14.

### Signaling pathway analysis

A signaling network of the top-ranking 1000 DM promoters was investigated using the SIGnaling Network Open Resource (SIGNOR) 2.0 15. Due to the large number of connections, the type of relation was selected to only include 'direct' interactions with a relaxed layout and a score of '0.0'.

## Results

### Sample clustering based on methylation data

**Figure 3:**
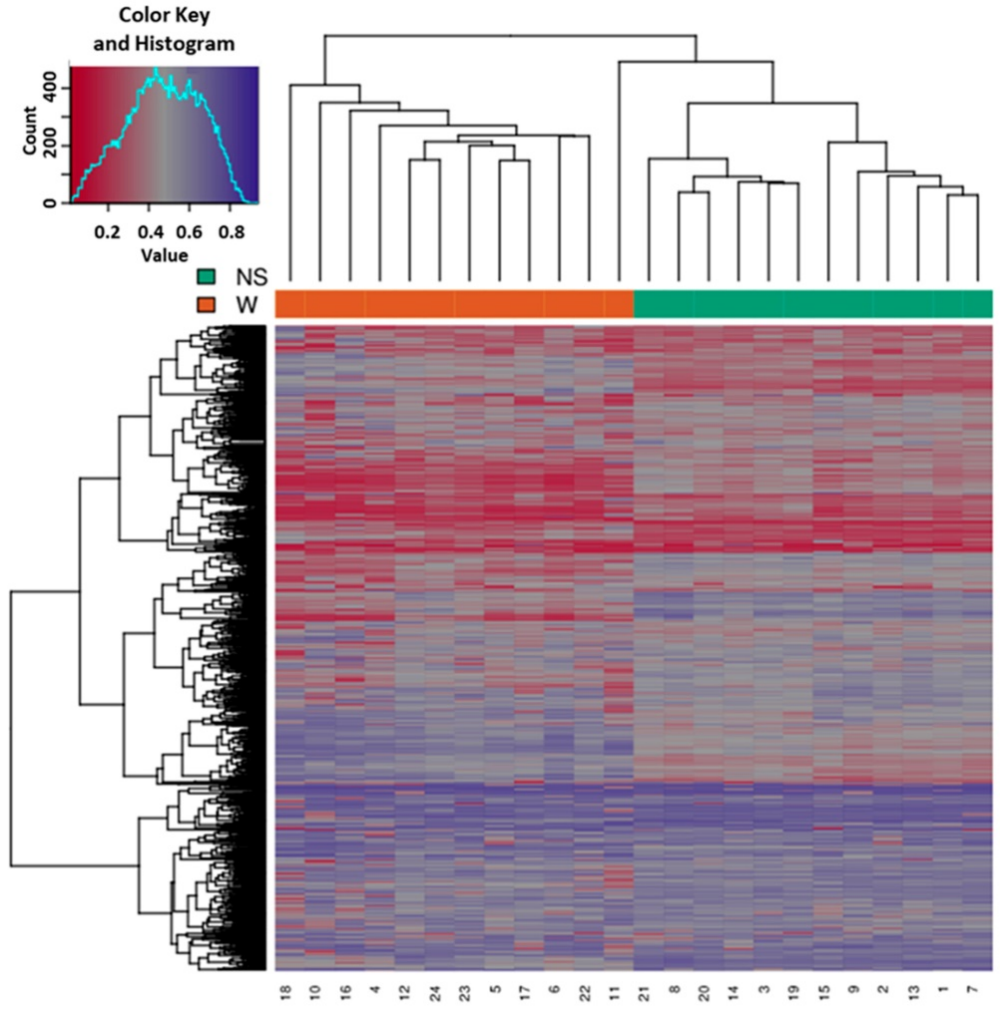
Heatmap showing the hierarchical clustering of samples displaying only the top-ranking 1000 most variable promoters with the highest variance across all samples. Clustering utilized complete linkage and Manhattan distance. The top x-axis shows the normal skin (NS) and wart (W) samples, while the bottom x-axis shows the patient identification number. Values of 0 (red color) and 1 (purple color) indicate decreased and increased methylation, respectively.

**Figure 4:**
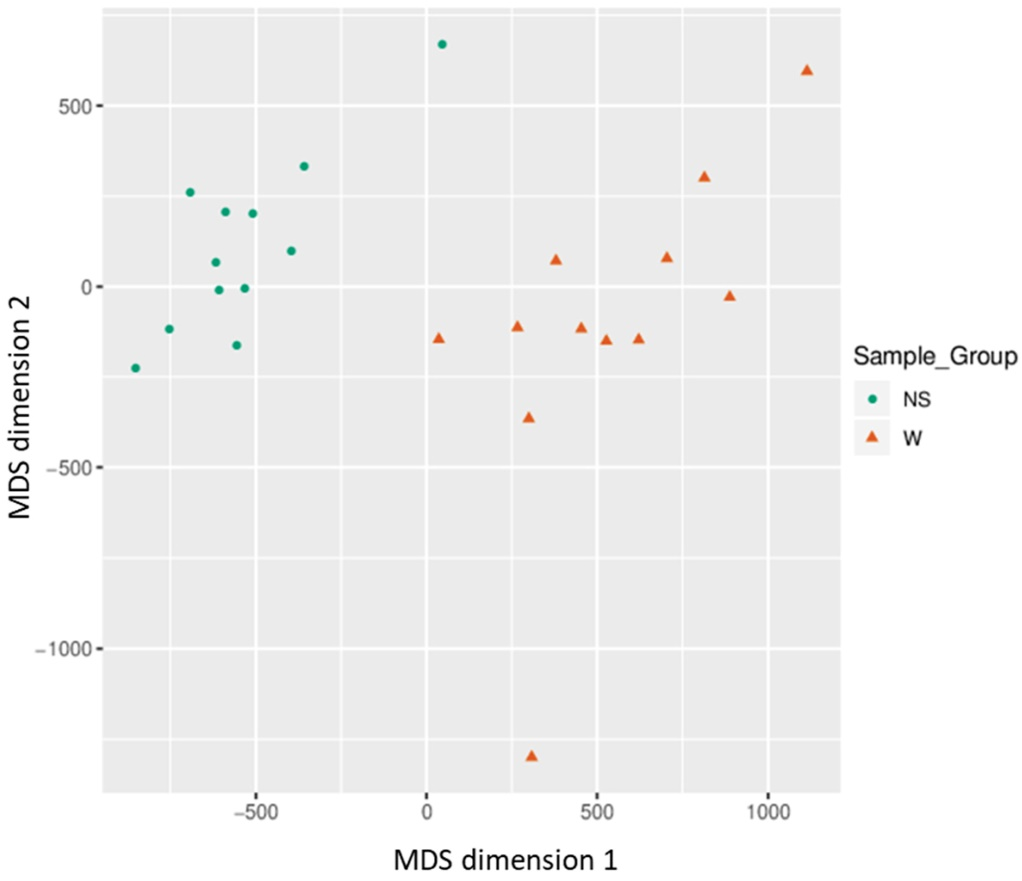
Two-dimensional scatterplot illustrating sample positions after the application of Kruskal's non-metric multidimensional scaling based on the matrix of average methylation and Manhattan distance.

Based on all methylation values of the top-ranking 1000 DM promoters, the 24 samples showed an expected clustering pattern, as samples with similar methylation patterns or phenotypes tended to cluster together (**Figure 3**). In addition, the dimension reduction test was applied to the dataset using multidimensional scaling (MDS) and principal component analysis (PCA) in order to inspect for a strong signal in the methylation values of the samples (**Figures 4** and** 5**). MDS and PCA confirmed that the difference between wart (W) and normal skin (NS) samples predominated the analysis.

### Differential methylation of promoters

**Figure 6:**
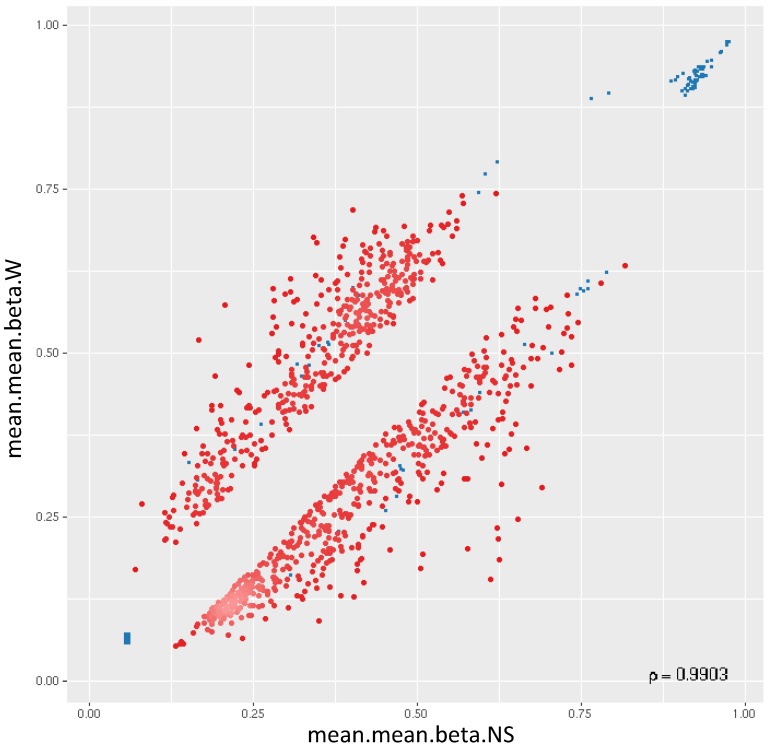
Two-dimensional scatterplot of the top-ranking 1000 DM promoters. The mean.mean β values of normal skin (NS) and warts (W) are shown on the x-axis and y-axis, respectively. The methylation β values range from 0 (unmethylated) to 1 (methylated).

**Figure 7:**
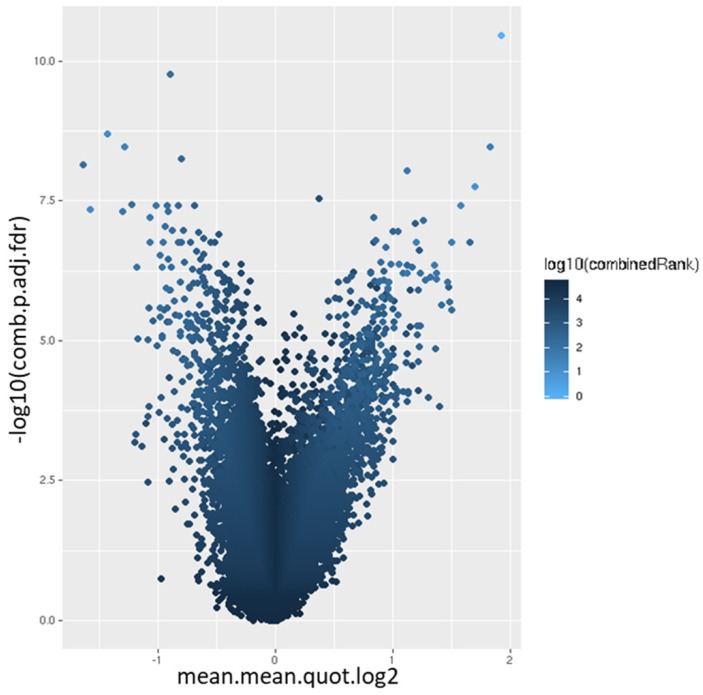
Volcano plot of the promoter differential methylation quantified by the log_2_ of the quotient in mean.mean methylation and adjusted combined p-value between warts (W) and normal skin (NS). The color scale represents the combined ranking.

44,929 genomic identifiers passed the quality control and pre-processing steps, including some identifiers that did not map to gene symbols or which were not assigned (NA). Genomic identifiers without symbols were then removed, leaving 27,790 with symbols. The list of DM promoters in warts was limited to the top-ranking 1000 DM promoters using the combined rank score. Using this scoring method, a total of 576 and 424 promoters were found to be hypomethylated and hypermethylated, respectively, in warts (W) compared to normal skin (NS), with a mean β difference =>0.064 and =< -0.064 and p-value =< 0.001 (adjusted p-value =<0.007) (**Figure 6**). Among the 576 hypomethylated promoters, the β difference ranged from -0.064 to -0.458, while the mean β difference ranged between 0.064 and 0.367 for the 424 hypermethylated promoters. The log_2_ of the quotient in methylation between warts and normal skin had a maximum value of 1.633 and minimum value of -1.924 (**Figure 7**). The top-ranking 100 DM promoters with the lowest combined rank score are shown in **Table 1**.

### Gene ontology enrichment analysis

**Figure 8:**
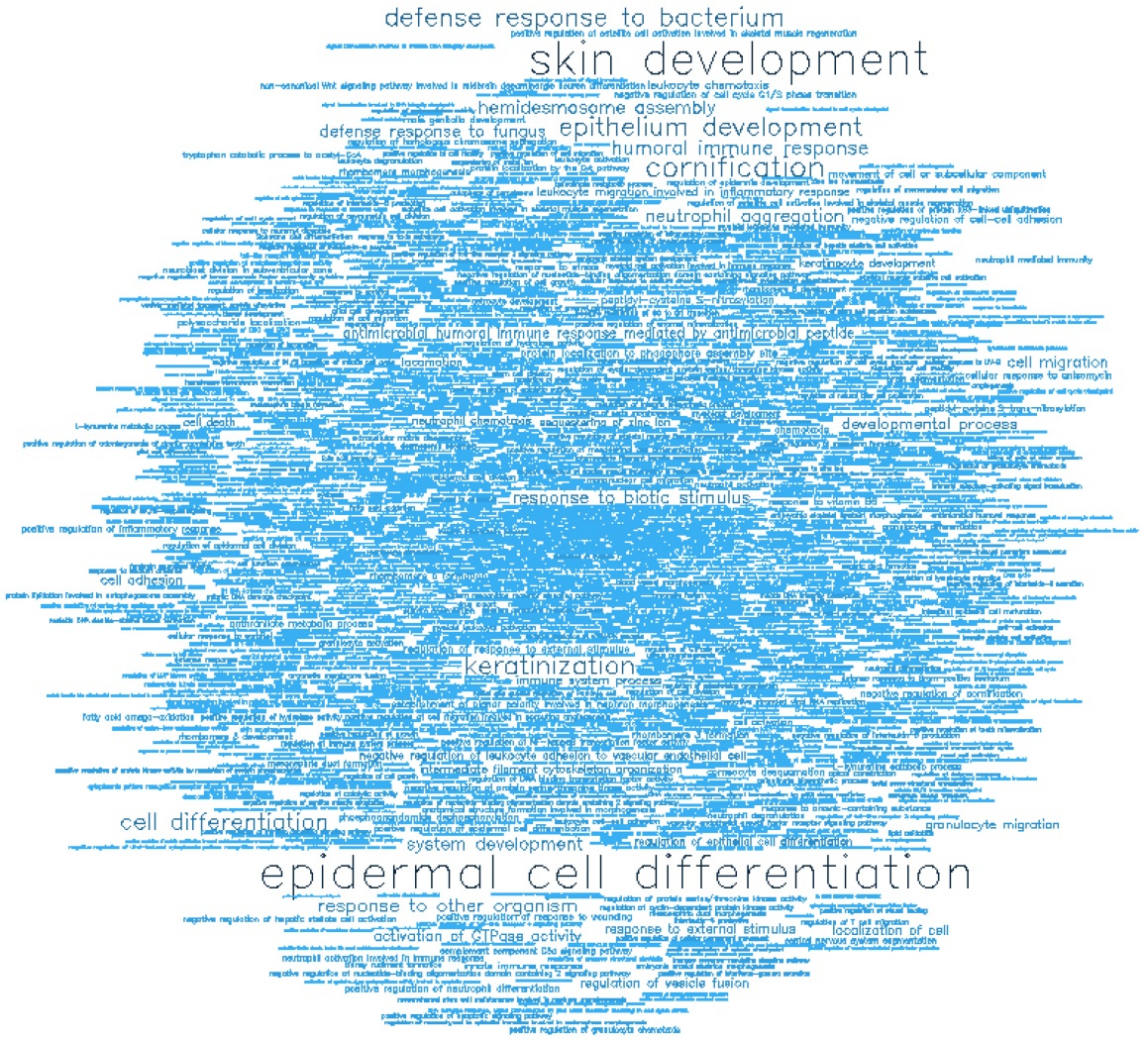
Word cloud illustrating the significant biological processes (BP) associated with the top-ranking 500 hypermethylated promoters.

**Figure 9:**
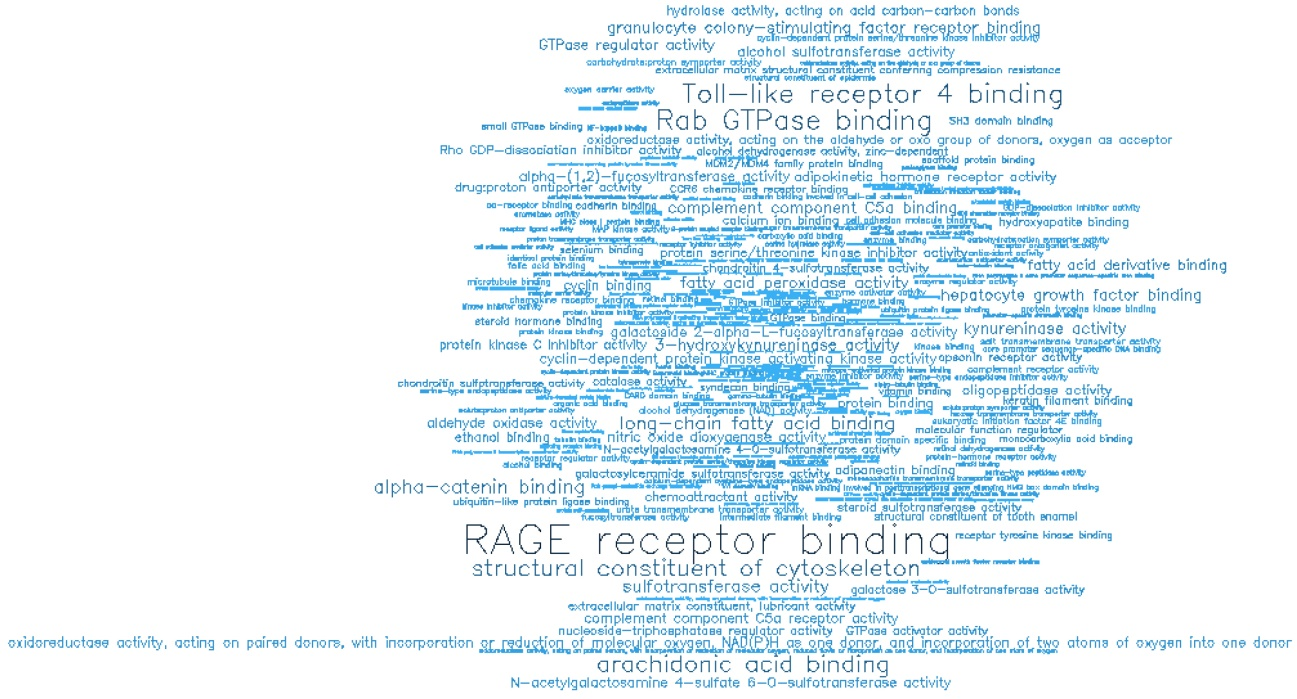
Word cloud illustrating the significant molecular functions (MF) associated with the top-ranking 500 hypermethylated promoters.

**Figure 10:**
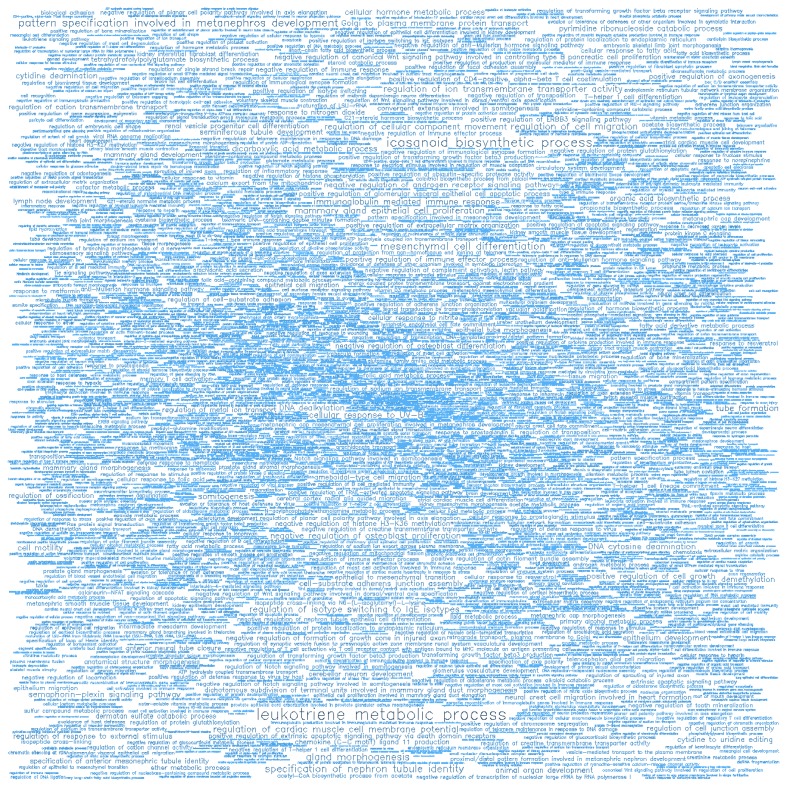
Word cloud illustrating the significant biological processes (BP) associated with the top-ranking 500 hypomethylated promoters.

**Figure 11:**
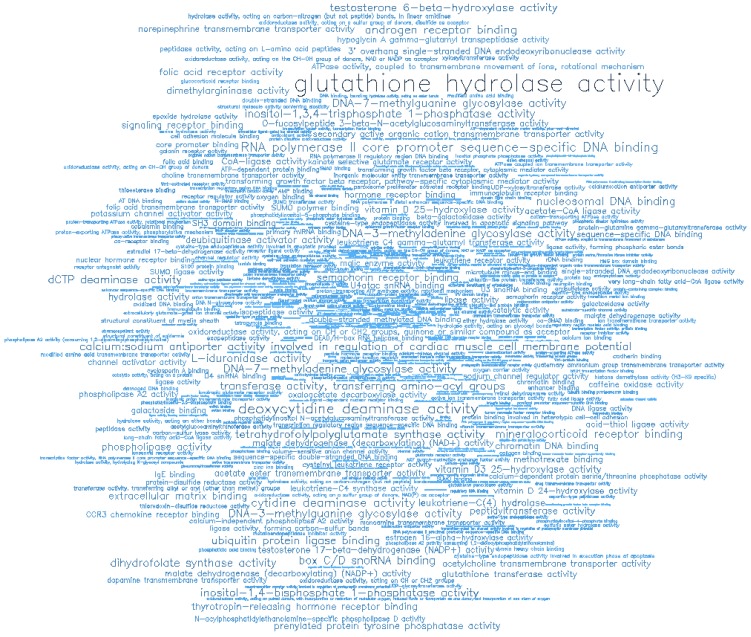
Word cloud illustrating the significant molecular functions (MF) associated with the top-ranking 500 hypomethylated promoters.

Gene ontology (GO) enrichment analysis of biological process (BP) and molecular function (MF) was conducted on the top-ranking 500 DM hypermethylated promoters (**Figure 8**, **Figure 9**, **Table 2**, and **Table 3**) and the top-ranking 500 DM hypomethylated promoters (**Figure 10**, **Figure 11**, **Table 4**, and **Table 5**).

### Pathway analysis

**Figure 12:**
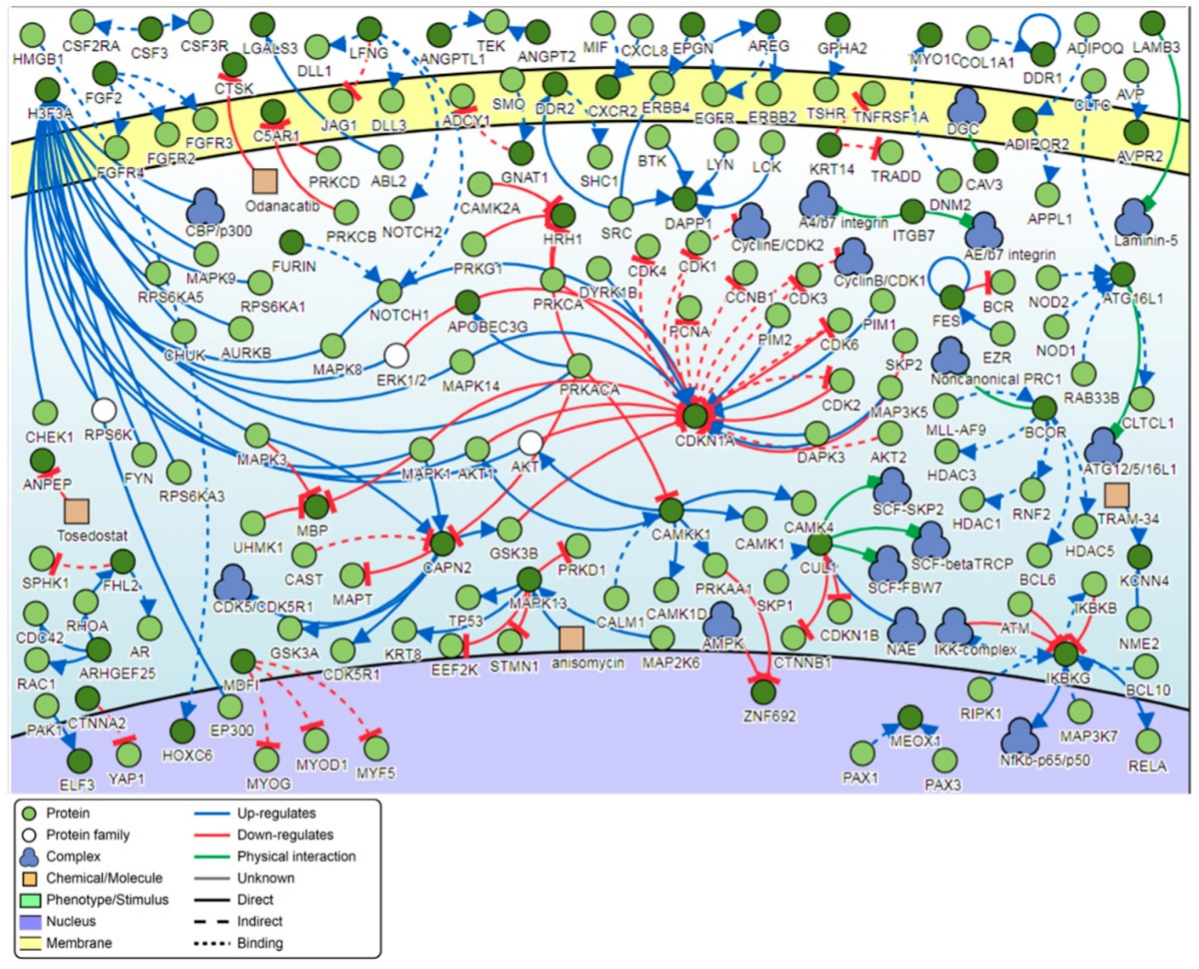
Pathway signaling network generated from the top-ranking 1000 DM promoters.

Signaling network analysis of the top-ranking 1000 DM promoters illustrated that several promoter genes were common regulators of this gene network, with a minimum of 7 direct connectivities each. These promoter genes include *H3F3A*, *CDKN1A*, *MAPK13*, *IKBKG*, *CAPN2*, *CAMKK1* and *CUL1* (**Figure 12**). Moreover, *H3F3A* was found to be the most common regulator when the signaling network analysis was carried out on the top 100 DM promoters.

## Discussion

To the best of the authors' knowledge, this is the first study to investigate the genome-wide changes in promoter methylation patterns associated with HPV-induced cutaneous warts. The present findings provide an exploratory analysis that creates clear lines of future research on this topic, especially with regard to validation studies involving larger sample sizes.

In the present study, the most differentially methylated (DM) promoter in warts compared to normal skin was found within the eukaryotic translation initiation factor 3 subunit E pseudogene 2 (*EIF3EP2*) gene, a pseudogene with no function or association previously reported in the literature. Likewise, little is known about the second most DM gene in warts, the chromosome 9 open reading frame 30 (*C9orf30*) pseudogene. In contrast, the third most DM gene is the protein-coding cysteinyl leukotriene receptor 1 (*CYSLTR1*) gene, which is normally involved in allergic and hypersensitive reactions 16. Variation in the *CYSLTR1* gene modulates asthma risk as well as adenoid hypertrophy progression, and it has been implicated in the disease outcome of colorectal, prostate, and squamous cell carcinoma 17-21. Moreover, *CYSLTR1* is highly expressed in the normal human skin epidermis, but its expression was found to be even higher in atopic dermatitis 22. **Table 2** depicts all the protein-coding genes containing DM promoters from among the top-ranking 100 listed in **Table 1**.

Among the protein-coding genes*, C10orf99* and *KRT6B* promoters exhibited high levels of differential methylation in warts. The chromosome 10 open reading frame 99 (*C10orf99*) gene encodes for an antimicrobial peptide that is widely expressed in the skin and digestive tract 23. In a pathologic context, *C10orf99* was determined to contribute to psoriasis development by promoting keratinocyte proliferation 24, 25. Likewise, the keratin 6B (*KRT6B*) gene encodes for a type II keratin that is normally present in mammalian epithelial cells and is rapidly induced in human keratinocytes after skin wounding 26. *KRT6B* has been identified as a potential biomarker for differentiating between lung adenocarcinoma and lung squamous cell carcinoma, and its increased expression is associated with lower disease-free survival rates in young breast cancer patients 27, 28. Mutations in the *KRT6B* gene result in an autosomal dominant skin disorder known as pachyonychia congenita, which involves plantar keratoderma and pain alongside thickened toenails 29. In contrast, two of the most differentially methylated protein-coding promoters, namely the kallikrein related peptidase 2 (*KLK2*) and Izumo sperm-egg fusion 1 (*IZUMO1*) genes, are integral for sperm function. *KLK2* over-expression has been associated with the promotion of prostate cancer cell growth 30.

As previously mentioned, the ephemeral nature of warts hints towards the involvement of an epigenetic component. Correspondingly, some of the most DM promoters were found within the laminin subunit alpha 4 (*LAMA4*) and H3 histone family member 3B (*H3F3B*) genes, which are responsible for cell differentiation and nucleosomal displacement, respectively 31, 32. In certain breast cancer subtypes, increased *LAMA4* expression was noted to contribute to the chromatin remodeling mechanisms that are a part of cancer progression 33. Moreover, atypical *HF3B* expression was reported to be associated with colorectal cancer and chondroblastoma 34, 35. On a similar note, epigenetic modifications have been linked to changes in metabolism in a number of different non-communicable diseases, including cancer and diabetes 36. In the present study, promoters were differentially methylated within the 17β-hydroxysteroid dehydrogenase type 14 (*HSD17B14*), leukotriene C4 synthase (*LTC4S*), folate receptor 3 (*FOLR3*), alcohol dehydrogenase 7 (*ADH7*), and adiponectin receptor 2 (*ADIPOR2*) genes that are involved in steroid, eicosanoid, folate, retinol, and glucose and lipid metabolism, respectively 37-41. Like the *CYSLTR1* gene, *LTC4S* polymorphisms were associated with asthma risk and drug responsiveness in different ethnic populations 42-45.

Pathway analysis demonstrated that the most common regulator among the top-ranking 1000 DM promoters was the H3 histone family member 3A (*H3F3A*) gene. Like the *H3F3B* gene, *H3F3A* encodes for a histone variant and is involved in transcriptional regulation 46. Aberrant *H3F3A* expression has been associated with the promotion of pediatric and adolescent cancers as well as lung cancer cell migration 46, 47. The second most common regulator was the cyclin dependent kinase inhibitor 1A (*CDKN1A*) gene, which is mostly involved in CDK2 inhibition and is a primary target of p53 activity 48. The *CDKN1A* gene was associated with better patient survival in HPV-related oropharyngeal squamous cell carcinoma 49. The third most common regulator in HPV-induced warts is the mitogen-activated protein kinase 13 (*MAPK13*) gene. *MAPK13* is a member of the MAP kinase family and functions to regulate cellular responses to a range of different stimuli, especially in the context of keratinocyte apoptosis and skin homeostasis 50. Analysis of genome-wide promoter methylation revealed that *MAPK13* was hypermethylated in the majority of primary and metastatic melanomas 51. *MAPK13* was also found to be hypermethylated in esophageal squamous cell carcinoma 52.

In summary, it is apparent that HPV-induced warts possess certain patterns of promoter methylation that could be responsible for their formation and maintenance. One limitation of the current study is that it is not possible at this stage to determine whether the differential methylation occurred as a result of the host cells' response to infection or due to HPV-driven processes responsible for wart formation and progression. Future research is required in order to assess the functional and clinical importance of the hypo- and hypermethylated promoter sites as well as to determine the exact nature of this differential methylation.

## Figures and Tables

**Figure 5:**
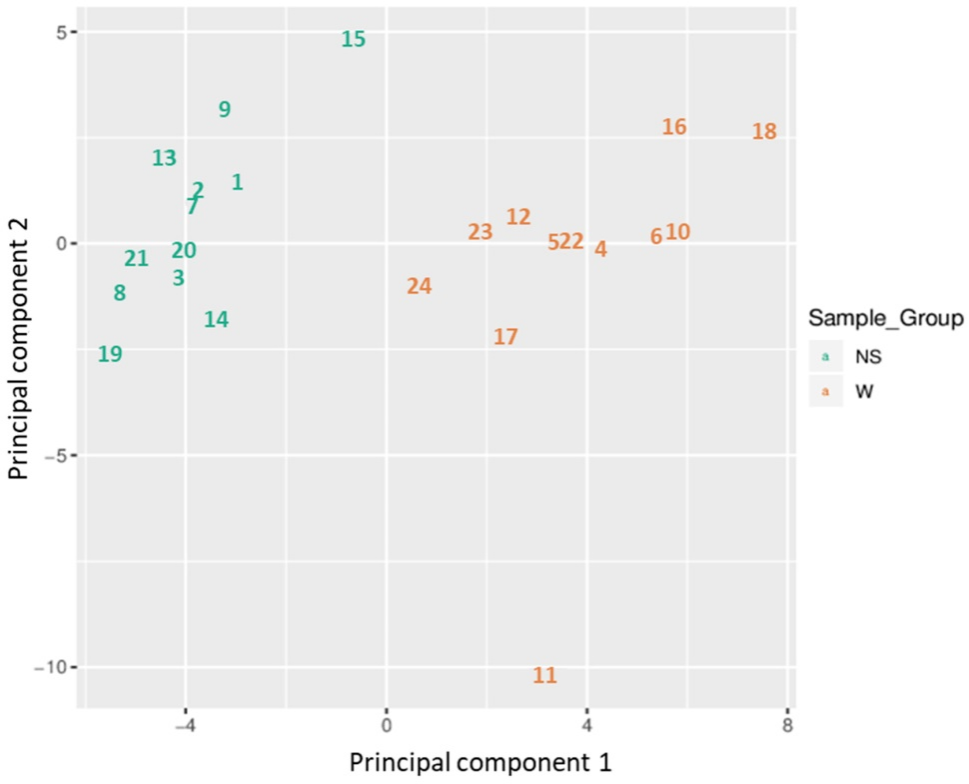
Two-dimensional scatterplot showing sample positions after principal component analysis.

**Figure 13:**
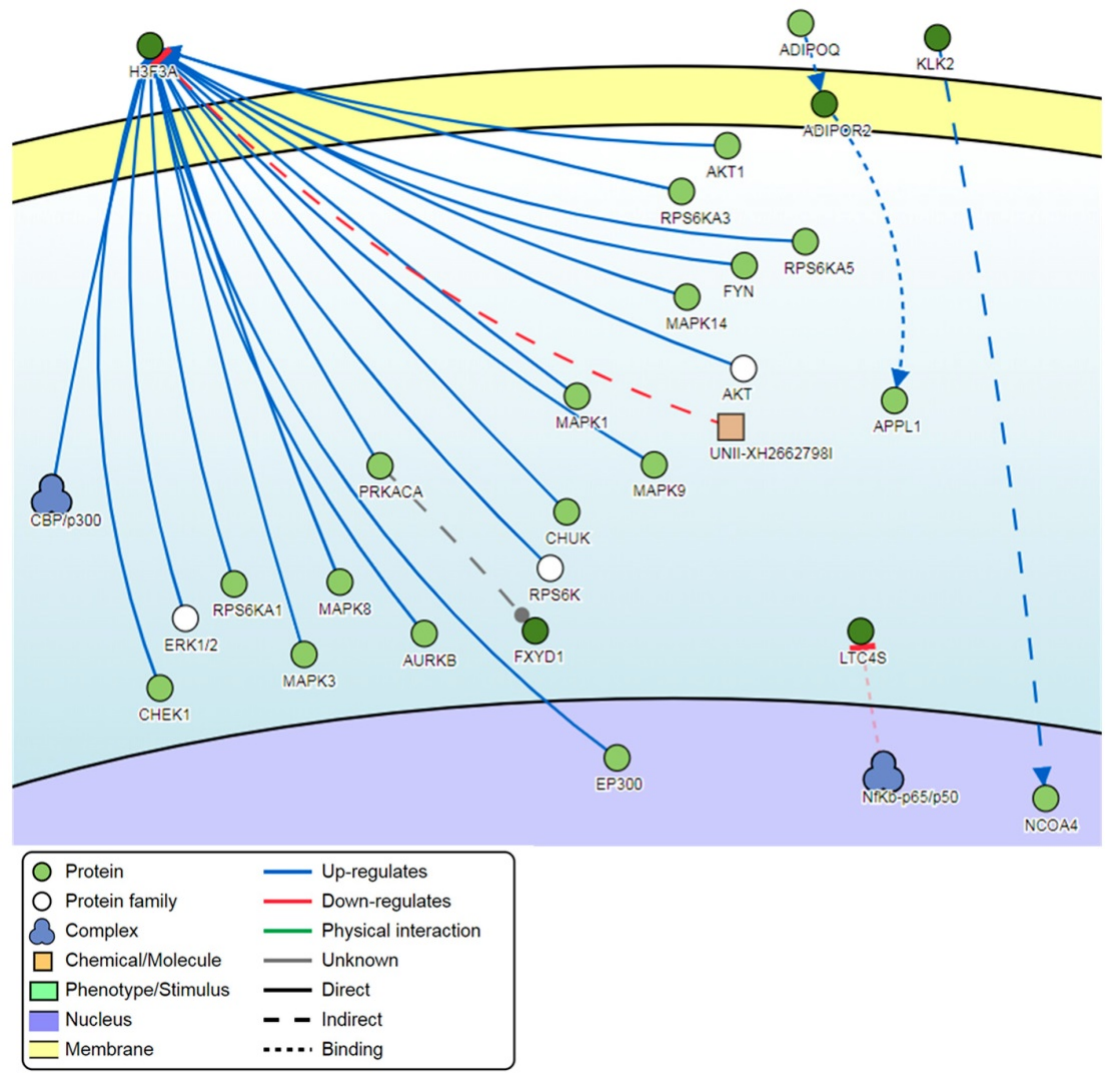
Pathway signaling network generated from the top-ranking 100 DM promoters.

**Table 1:** The 100 top-ranking promoters based on combined ranking score.

Gene	Gene symbol	Category	RNA class	Chromosome	mean.mean β value (NS)	mean.mean β value (W)	mean.mean β value diff. (W-NS)	mean.mean. quot.log_2_	comb.p.val	comb.p.adj. (FDR)	Combined rank
ENSG00000224674	EIF3EP2	Pseudogene		2	0.611	0.154	-0.458	-1.924	7.832E-16	3.519E-11	1
ENSG00000263368	AC069366.1	Pseudogene	antisense	17	0.206	0.573	0.367	1.431	1.336E-13	2.001E-09	14
ENSG00000173198	CYSLTR1	Protein coding		X	0.166	0.520	0.353	1.580	1.671E-11	4.415E-08	17
ENSG00000266228	MIR3611	RNA gene	miRNA	10	0.403	0.128	-0.275	-1.584	1.083E-11	3.744E-08	27
ENSG00000267125	AC012615.3	RNA gene		19	0.192	0.465	0.273	1.280	3.531E-13	3.410E-09	29
ENSG00000270808	AC022400.4	Pseudogene	lncRNA	10	0.691	0.295	-0.396	-1.202	1.686E-10	1.756E-07	40
ENSG00000241114	AC008280.2	Pseudogene		2	0.383	0.129	-0.254	-1.503	1.876E-10	1.756E-07	47
ENSG00000272156	AC008280.3	RNA gene		2	0.383	0.129	-0.254	-1.503	1.876E-10	1.756E-07	47
ENSG00000207258	RF00019	RNA gene	Y RNA	1	0.508	0.192	-0.315	-1.356	6.373E-10	4.522E-07	62
ENSG00000226545	AL357552.1	Pseudogene		1	0.508	0.192	-0.315	-1.356	6.373E-10	4.522E-07	62
ENSG00000270002	AC022028.2	RNA gene		10	0.458	0.199	-0.259	-1.056	5.656E-10	4.246E-07	70
ENSG00000227096	HMGB3P8	Pseudogene		10	0.653	0.246	-0.408	-1.376	1.138E-09	6.141E-07	82
ENSG00000250532	AC021180.1	RNA gene		4	0.621	0.233	-0.388	-1.378	1.576E-09	7.956E-07	89
ENSG00000254653	AC024475.1	RNA gene		11	0.228	0.440	0.212	1.071	1.411E-10	1.756E-07	99
ENSG00000265503	MIR1269B	RNA gene	miRNA	17	0.346	0.141	-0.205	-1.239	1.140E-09	6.141E-07	109
ENSG00000238024	DDX39BP2	Pseudogene		6	0.326	0.124	-0.202	-1.323	1.391E-09	7.248E-07	113
ENSG00000273044	AL022334.2	RNA gene		22	0.243	0.481	0.238	0.956	1.766E-10	1.756E-07	119
ENSG00000234105	AC009468.2	RNA gene		7	0.576	0.307	-0.269	-0.961	3.100E-09	1.151E-06	121
ENSG00000188373	C10orf99	Protein coding		10	0.400	0.202	-0.198	-0.982	5.786E-10	4.262E-07	124
ENSG00000271597	AC112230.1	Pseudogene	lncRNA	2	0.306	0.594	0.287	0.933	3.019E-09	1.151E-06	136
ENSG00000271265	AL355297.3	RNA gene	lncRNA	6	0.347	0.667	0.320	0.924	1.317E-11	3.856E-08	145
ENSG00000244286	ITGB5-AS1	RNA gene	ncRNA	3	0.202	0.393	0.190	1.223	8.912E-12	3.640E-08	152
ENSG00000226403	AL392089.1	RNA gene		9	0.080	0.269	0.189	1.633	1.086E-12	6.969E-09	154
ENSG00000234936	AC010883.1	RNA gene		2	0.288	0.498	0.210	0.909	2.020E-11	4.908E-08	158
ENSG00000203527	Z99756.1	RNA gene	ncRNA	22	0.385	0.198	-0.187	-0.906	5.290E-09	1.674E-06	161
ENSG00000242147	AL365356.5	RNA gene	ncRNA	10	0.334	0.148	-0.186	-1.263	3.506E-11	7.161E-08	166
ENSG00000270781	AC091133.5	Pseudogene		17	0.416	0.219	-0.197	-0.896	4.980E-10	3.925E-07	170
ENSG00000250282	AC002401.2	RNA gene		17	0.225	0.443	0.217	0.894	3.488E-09	1.234E-06	174
ENSG00000255158	AC131934.1	RNA gene		11	0.299	0.590	0.291	0.977	1.016E-08	2.625E-06	174
ENSG00000232486	AL592437.2	Pseudogene		9	0.666	0.354	-0.312	-0.892	1.930E-09	8.758E-07	175
ENSG00000262067	AC120057.1	Pseudogene	lncRNA	17	0.505	0.171	-0.333	-1.505	1.139E-08	2.816E-06	181
ENSG00000266258	LINC01909	RNA gene	ncRNA	18	0.629	0.299	-0.330	-1.048	1.479E-08	3.408E-06	195
ENSG00000257496	AC025031.1	RNA gene		12	0.217	0.397	0.180	0.982	1.649E-08	3.703E-06	200
ENSG00000185479	KRT6B	Protein-coding		12	0.340	0.166	-0.174	-1.045	7.004E-11	1.124E-07	216
ENSG00000270255	AC009884.2	Pseudogene		8	0.279	0.529	0.250	0.900	2.221E-08	4.587E-06	217
ENSG00000167751	KLK2	Protein coding		19	0.328	0.136	-0.192	-1.214	2.772E-08	5.463E-06	227
ENSG00000268518	AC020909.2	RNA gene		19	0.432	0.238	-0.194	-0.839	2.897E-11	6.197E-08	229
ENSG00000243795	LINC02044	RNA gene	ncRNA	3	0.387	0.663	0.276	0.825	1.229E-11	3.856E-08	246
ENSG00000267632	AC067852.3	RNA gene	lncRNA	17	0.402	0.719	0.316	0.821	1.561E-10	1.756E-07	254
ENSG00000259265	AC027088.3	RNA gene		15	0.362	0.195	-0.167	-0.918	2.209E-08	4.587E-06	260
ENSG00000264733	MIR4718	RNA gene	miRNA	16	0.342	0.176	-0.166	-0.922	1.674E-09	8.164E-07	263
ENSG00000253630	AC026407.1	Pseudogene	antisense	5	0.537	0.301	-0.236	-0.815	1.148E-09	6.141E-07	264
ENSG00000228918	LINC01344	RNA gene	ncRNA	1	0.180	0.346	0.166	0.908	4.232E-10	3.475E-07	264
ENSG00000232878	DPYD-AS1	RNA gene	ncRNA	1	0.572	0.387	-0.185	-0.815	4.216E-08	7.523E-06	265
ENSG00000112769	LAMA4	Protein coding		6	0.325	0.512	0.187	0.810	3.888E-08	7.072E-06	269
ENSG00000237126	AC073254.1	RNA gene		2	0.368	0.202	-0.166	-0.835	1.684E-08	3.745E-06	270
ENSG00000256746	AC018410.1	RNA gene	ncRNA	11	0.344	0.536	0.192	0.807	2.084E-09	9.002E-07	271
ENSG00000232560	C21orf37	RNA gene	ncRNA	21	0.300	0.495	0.195	0.805	5.048E-08	8.338E-06	274
ENSG00000198796	ALPK2	Protein coding		18	0.165	0.329	0.163	0.924	1.006E-08	2.622E-06	286
ENSG00000185432	METTL7A	Protein coding		12	0.389	0.673	0.283	0.795	7.318E-13	5.480E-09	286
ENSG00000087076	HSD17B14	Protein coding		19	0.145	0.346	0.201	1.168	5.925E-08	9.251E-06	287
ENSG00000239255	AC007620.1	Pseudogene		3	0.347	0.575	0.227	1.085	6.353E-08	9.775E-06	292
ENSG00000230403	LINC01066	RNA gene	ncRNA	13	0.475	0.302	-0.173	-0.902	8.129E-08	1.192E-05	306
ENSG00000132475	H3F3B	Protein coding		17	0.173	0.358	0.184	1.003	8.349E-08	1.218E-05	308
ENSG00000258274	AC012085.2	RNA gene	ncRNA	12	0.415	0.624	0.208	0.785	6.143E-11	1.062E-07	308
ENSG00000244167	AC005532.2	Pseudogene	lncRNA	7	0.488	0.281	-0.207	-0.775	4.380E-08	7.657E-06	324
ENSG00000266740	MIR4708	RNA gene	miRNA	14	0.240	0.416	0.177	0.771	4.844E-10	3.887E-07	328
ENSG00000258657	AL136018.1	RNA gene		14	0.448	0.234	-0.213	-0.946	1.163E-07	1.588E-05	329
ENSG00000186715	MST1L	Protein coding		1	0.300	0.145	-0.156	-1.006	6.774E-11	1.124E-07	335
ENSG00000253543	AC083923.1	Pseudogene		8	0.277	0.121	-0.156	-1.127	6.441E-10	4.522E-07	339
ENSG00000261095	AC136285.1	RNA gene	ncRNA	16	0.487	0.272	-0.215	-0.958	1.368E-07	1.803E-05	341
ENSG00000213316	LTC4S	Protein coding		5	0.211	0.365	0.154	1.075	1.025E-08	2.631E-06	348
ENSG00000267299	AC011444.3	RNA gene		19	0.141	0.300	0.159	0.752	2.936E-08	5.687E-06	352
ENSG00000234502	FYTTD1P1	Pseudogene		9	0.361	0.180	-0.182	-0.969	1.678E-07	2.118E-05	356
ENSG00000265666	RARA-AS1	RNA gene	ncRNA	17	0.189	0.339	0.151	0.868	1.088E-07	1.501E-05	370
ENSG00000182264	IZUMO1	Protein coding		19	0.308	0.468	0.160	0.737	3.157E-08	5.910E-06	376
ENSG00000254113	AC090193.2	RNA gene		8	0.243	0.419	0.177	0.736	1.371E-08	3.276E-06	378
ENSG00000204933	CD177P1	Pseudogene		19	0.375	0.607	0.232	0.733	2.798E-09	1.103E-06	382
ENSG00000110203	FOLR3	Protein coding		11	0.536	0.357	-0.180	-0.746	1.990E-07	2.311E-05	387
ENSG00000266964	FXYD1	Protein coding		19	0.299	0.452	0.154	0.731	3.068E-09	1.151E-06	391
ENSG00000221857	AC020907.2	RNA gene		19	0.299	0.452	0.154	0.731	3.068E-09	1.151E-06	391
ENSG00000213417	KRTAP2-4	Protein coding		17	0.471	0.309	-0.163	-0.855	2.328E-07	2.604E-05	401
ENSG00000254853	AP004247.1	Pseudogene		11	0.247	0.100	-0.147	-1.221	3.631E-09	1.265E-06	410
ENSG00000283664; ENSG00000265375	MIR4679-1; MIR4679-2	RNA gene	miRNA	10	0.353	0.589	0.236	0.722	1.858E-10	1.756E-07	410
ENSG00000261257	AP000821.1	RNA gene	lncRNA	11	0.394	0.543	0.149	0.746	2.524E-07	2.757E-05	411
ENSG00000204880	KRTAP4-8	Protein coding		17	0.356	0.198	-0.158	-0.823	2.945E-07	3.114E-05	425
ENSG00000215930	MIR942	RNA gene	miRNA	1	0.410	0.266	-0.144	-0.782	8.361E-09	2.305E-06	427
ENSG00000271680	AC098935.2	Pseudogene	antisense	1	0.244	0.100	-0.144	-1.211	4.741E-08	7.978E-06	428
ENSG00000258380	AL356805.1	RNA gene		14	0.292	0.435	0.144	1.043	2.010E-08	4.354E-06	432
ENSG00000249717	AC110760.2	RNA gene	ncRNA	4	0.480	0.694	0.213	0.707	5.817E-08	9.171E-06	436
ENSG00000265462; ENSG00000266758	MIR3680-1; MIR3680-2	RNA gene	miRNA	16	0.383	0.630	0.247	0.705	8.156E-10	5.161E-07	438
ENSG00000263361	MIR378H	RNA gene	miRNA	5	0.411	0.268	-0.143	-0.731	7.162E-08	1.080E-05	443
ENSG00000249483	AC026726.1	RNA gene	lncRNA	5	0.114	0.257	0.142	0.852	1.524E-08	3.459E-06	446
ENSG00000227735	CYCSP5	Pseudogene	antisense	1	0.212	0.070	-0.142	-1.478	2.842E-09	1.110E-06	449
ENSG00000267130	AC008738.2	RNA gene		19	0.163	0.310	0.146	0.698	3.087E-08	5.835E-06	449
ENSG00000269480	AC020913.2	RNA gene		19	0.388	0.226	-0.162	-0.755	4.211E-07	3.933E-05	481
ENSG00000260673	AL034376.1	RNA gene		6	0.392	0.254	-0.139	-0.703	8.046E-08	1.187E-05	482
ENSG00000261392	AC087190.2	RNA gene		16	0.735	0.481	-0.255	-0.681	2.657E-07	2.869E-05	488
ENSG00000196344	ADH7	Protein coding		4	0.290	0.152	-0.138	-0.936	2.488E-07	2.740E-05	488
ENSG00000170454	KRT75	Protein coding		12	0.467	0.293	-0.175	-0.729	4.448E-07	4.087E-05	489
ENSG00000254175	IGLVI-42	Pseudogene		22	0.232	0.095	-0.137	-1.201	1.475E-10	1.756E-07	498
ENSG00000254073	IGLVVII-41-1	Pseudogene		22	0.232	0.095	-0.137	-1.201	1.475E-10	1.756E-07	498
ENSG00000253947	AC008705.1	RNA gene		5	0.393	0.582	0.189	0.677	1.507E-08	3.438E-06	498
ENSG00000275874	PICSAR	RNA gene	ncRNA	21	0.467	0.318	-0.150	-0.675	2.541E-07	2.757E-05	503
ENSG00000233930	KRTAP5-AS1	RNA gene	ncRNA	11	0.162	0.298	0.136	0.944	1.138E-08	2.816E-06	503
ENSG00000188100	FAM25A	Protein coding		10	0.389	0.254	-0.135	-0.688	1.426E-07	1.862E-05	509
ENSG00000261078	AC009118.1	RNA gene		16	0.250	0.115	-0.135	-1.008	2.220E-08	4.587E-06	513
ENSG00000259195	AC021739.1	Pseudogene		15	0.284	0.149	-0.134	-0.927	4.219E-08	7.523E-06	519
ENSG00000260905	AC009021.1	RNA gene		16	0.616	0.384	-0.232	-0.667	8.523E-08	1.235E-05	523
ENSG00000006831	ADIPOR2	Protein coding		12	0.721	0.501	-0.220	-0.667	1.007E-07	1.410E-05	527

**Table 2:** Function and clinical relevance of the protein-coding genes containing the most differentially methylated promoters in warts

Gene symbol	Gene name	Main physiological function
*CYSLTR1*	Cysteinyl leukotriene receptor 1	Mediates bronchoconstriction
*C10orf99*	Chromosome 10 Open Reading Frame 99	Mediates recruitment of lymphocytes to epithelia
*KRT6B*	Keratin 6B	Epithelial wound repair and inflammation
*KLK2*	Kallikrein Related Peptidase 2	Sperm liquefication
*LAMA4*	Laminin Subunit Alpha 4	Cell adhesion, differentiation, and migration
*ALPK2*	Alpha Kinase 2	Unknown
*METTL7A*	Methyltransferase Like 7A	Unknown
*HSD17B14*	17β-Hydroxysteroid dehydrogenase type 14	Steroid metabolism
*H3F3B*	H3 Histone Family Member 3B	Found at sites of nucleosomal displacement
*MST1L*	Macrophage Stimulating 1 Like	Unknown
*LTC4S*	Leukotriene C4 Synthase	Involved in cysteinyl leukotriene biosynthesis
*IZUMO1*	Izumo sperm-egg fusion 1	Essential for fusion and binding of sperm and egg
*FOLR3*	Folate receptor 3	Mediate delivery of 5-methyltetrahydrofolate to cell interior
*FXYD1*	FXYD Domain Containing Ion Transport Regulator 1	Regulates ion channel activity
*KRTAP2-4*	Keratin Associated Protein 2-4	Involved in hair formation
*KRTAP4-8*	Keratin Associated Protein 4-8	Involved in hair formation
*ADH7*	Alcohol dehydrogenase 7	Functions in retinoic acid synthesis
*KRT75*	Keratin 75	Involved in hair and nail formation
*FAM25A*	Family with sequence similarity 25 member A	Unknown
*ADIPOR2*	Adiponectin receptor 2	Regulates glucose and lipid metabolism

**Table 3:** GO enrichment analysis showing the significant biological processes (BP) of the top 500 hypermethylated promoters.

GOMFID	P-value	Odds ratio	ExpCount	Count	Size	Term
GO:0009913	0	11.3215	2.1081	19	328	epidermal cell differentiation
GO:0043588	0	9.3276	2.6737	20	409	skin development
GO:0070268	0	14.8063	0.7126	9	110	cornification
GO:0031424	0	13.9409	0.664	8	111	keratinization
GO:0060429	0	3.7163	8.1779	25	1251	epithelium development
GO:0042742	0	8.0356	1.5624	11	239	defense response to bacterium
GO:0030154	0	2.5597	26.423	49	4042	cell differentiation
GO:0006959	0	6.7129	1.4905	9	228	humoral immune response
GO:0051707	0	3.5529	5.4585	17	835	response to other organism
GO:0070488	0	Inf	0.0131	2	2	neutrophil aggregation
GO:0031581	0	58.5596	0.0719	3	11	hemidesmosome assembly
GO:0009607	1e-04	3.3622	5.7461	17	879	response to biotic stimulus
GO:0048731	1e-04	2.1729	30.6198	50	4684	system development
GO:0050832	1e-04	17.986	0.2549	4	39	defense response to fungus
GO:0032502	2e-04	2.0271	40.0921	59	6133	developmental process
GO:0016477	2e-04	2.639	9.1062	21	1393	cell migration
GO:0090630	3e-04	9.5433	0.5753	5	88	activation of GTPase activity
GO:0061844	5e-04	11.8647	0.3726	4	57	antimicrobial humoral immune response mediated by antimicrobial peptide
GO:0009605	8e-04	2.5057	8.646	19	1419	response to external stimulus
GO:0051674	8e-04	2.378	10.0018	21	1530	localization of cell
GO:0007155	9e-04	2.4699	8.6421	19	1322	cell adhesion
GO:0031338	0.001	9.819	0.4445	4	68	regulation of vesicle fusion
GO:0097530	0.001	7.0599	0.7648	5	117	granulocyte migration
GO:0002376	0.0014	2.0026	18.1078	31	2770	immune system process
GO:0002523	0.0018	38.6782	0.0654	2	10	leukocyte migration involved in inflammatory response
GO:0030595	0.0019	4.9654	1.2943	6	198	leukocyte chemotaxis
GO:0040011	0.002	2.1602	11.4792	22	1756	locomotion
GO:1904995	0.0022	34.3786	0.0719	2	11	negative regulation of leukocyte adhesion to vascular endothelial cell
GO:0045104	0.0023	12.6396	0.2615	3	40	intermediate filament cytoskeleton organization
GO:0030593	0.0025	7.5626	0.5687	4	87	neutrophil chemotaxis
GO:0003334	0.0027	30.9389	0.0784	2	12	keratinocyte development
GO:0032119	0.0027	30.9389	0.0784	2	12	sequestering of zinc ion
GO:0008219	0.0029	2.0056	14.1398	25	2163	cell death
GO:0030856	0.003	5.4424	0.9806	5	150	regulation of epithelial cell differentiation
GO:0018119	0.0032	28.1246	0.085	2	13	peptidyl-cysteine S-nitrosylation
GO:0034497	0.0032	28.1246	0.085	2	13	protein localization to phagophore assembly site
GO:0032101	0.0034	2.6778	4.8571	12	743	regulation of response to external stimulus
GO:0022408	0.0036	5.2242	1.0198	5	156	negative regulation of cell-cell adhesion
GO:0006928	0.0045	1.979	13.0285	23	1993	movement of cell or subcellular component
GO:0006935	0.0049	2.8124	3.8177	10	584	chemotaxis
GO:0045087	0.0051	2.4353	5.7853	13	885	innate immune response
GO:0003336	0.0065	Inf	0.0065	1	1	corneocyte desquamation
GO:0021593	0.0065	Inf	0.0065	1	1	rhombomere morphogenesis
GO:0021660	0.0065	Inf	0.0065	1	1	rhombomere 3 formation
GO:0021666	0.0065	Inf	0.0065	1	1	rhombomere 5 formation
GO:0033037	0.0065	Inf	0.0065	1	1	polysaccharide localization
GO:0034516	0.0065	Inf	0.0065	1	1	response to vitamin B6
GO:0035644	0.0065	Inf	0.0065	1	1	phosphoanandamide dephosphorylation
GO:0043420	0.0065	Inf	0.0065	1	1	anthranilate metabolic process
GO:0045660	0.0065	Inf	0.0065	1	1	positive regulation of neutrophil differentiation
GO:0072046	0.0065	Inf	0.0065	1	1	establishment of planar polarity involved in nephron morphogenesis
GO:0072740	0.0065	Inf	0.0065	1	1	cellular response to anisomycin
GO:1905716	0.0065	Inf	0.0065	1	1	negative regulation of cornification
GO:0006950	0.008	1.6938	24.6188	36	3766	response to stress
GO:1903036	0.0081	7.7836	0.4118	3	63	positive regulation of response to wounding
GO:0050729	0.0082	5.354	0.791	4	121	positive regulation of inflammatory response
GO:0030539	0.0082	16.2749	0.1373	2	21	male genitalia development
GO:1902807	0.0087	5.2634	0.8041	4	123	negative regulation of cell cycle G1/S phase transition
GO:0045606	0.0098	14.7231	0.1504	2	23	positive regulation of epidermal cell differentiation
GO:0001775	0.0099	2.0563	8.459	16	1294	cell activation

**Table 4:** GO enrichment analysis showing the significant molecular functions (MF) of the top 500 hypermethylated promoters.

GOMFID	P-value	Odds ratio	ExpCount	Count	Size	Term
GO:0050786	0	99.375	0.0655	4	11	RAGE receptor binding
GO:0017137	1e-04	7.2337	1.0653	7	179	Rab GTPase binding
GO:0035662	1e-04	340.8367	0.0179	2	3	Toll-like receptor 4 binding
GO:0050544	3e-04	113.5986	0.0298	2	5	arachidonic acid binding
GO:0005200	4e-04	8.9173	0.613	5	103	structural constituent of cytoskeleton
GO:0045294	0.0019	37.8526	0.0655	2	11	alpha-catenin binding
GO:0036041	0.0022	34.0653	0.0714	2	12	long-chain fatty acid binding
GO:0008146	0.0035	10.7307	0.3035	3	51	sulfotransferase activity
GO:0001856	0.006	Inf	0.006	1	1	complement component C5a binding
GO:0005130	0.006	Inf	0.006	1	1	granulocyte colony-stimulating factor receptor binding
GO:0030429	0.006	Inf	0.006	1	1	kynureninase activity
GO:0036458	0.006	Inf	0.006	1	1	hepatocyte growth factor binding
GO:0047888	0.006	Inf	0.006	1	1	fatty acid peroxidase activity
GO:0061981	0.006	Inf	0.006	1	1	3-hydroxykynureninase activity
GO:1901567	0.0096	14.7995	0.1488	2	25	fatty acid derivative binding

**Table 5:** GO enrichment analysis showing the significant biological processes (BP) of the top 500 hypomethylated promoters.

GOMFID	P-value	Odds ratio	ExpCount	Count	Size	Term
GO:1901750	0	102.821	0.0789	4	8	leukotriene D4 biosynthetic process
GO:0006751	0	82.2519	0.0888	4	9	glutathione catabolic process
GO:0006691	0	21.5282	0.2861	5	29	leukotriene metabolic process
GO:0046456	1e-04	12.589	0.4538	5	46	icosanoid biosynthetic process
GO:0051572	3e-04	203.1707	0.0296	2	3	negative regulation of histone H3-K4 methylation
GO:0006575	4e-04	4.9708	1.7363	8	176	cellular modified amino acid metabolic process
GO:0072268	6e-04	101.5793	0.0395	2	4	pattern specification involved in metanephros development
GO:0048762	9e-04	4.3203	1.9829	8	201	mesenchymal cell differentiation
GO:0072081	9e-04	67.7154	0.0493	2	5	specification of nephron tubule identity
GO:0022612	0.0012	5.4916	1.1739	6	119	gland morphogenesis
GO:0040012	0.0012	2.2988	9.4409	20	957	regulation of locomotion
GO:0030155	0.0015	2.5831	6.2347	15	632	regulation of cell adhesion
GO:0030334	0.0016	2.3435	8.2867	18	840	regulation of cell migration
GO:0051893	0.0018	8.3709	0.5229	4	53	regulation of focal adhesion assembly
GO:0017144	0.0018	2.3773	7.6948	17	780	drug metabolic process
GO:0048293	0.002	40.6244	0.0691	2	7	regulation of isotype switching to IgE isotypes
GO:0086036	0.002	40.6244	0.0691	2	7	regulation of cardiac muscle cell membrane potential
GO:0032412	0.002	3.7493	2.269	8	230	regulation of ion transmembrane transporter activity
GO:0033598	0.0023	12.7584	0.2664	3	27	mammary gland epithelial cell proliferation
GO:0071493	0.0026	33.8516	0.0789	2	8	cellular response to UV-B
GO:1902041	0.0027	7.455	0.582	4	59	regulation of extrinsic apoptotic signaling pathway via death domain receptors
GO:0035148	0.003	4.523	1.4107	6	143	tube formation
GO:0016064	0.0031	5.4155	0.9865	5	100	immunoglobulin mediated immune response
GO:0033689	0.0033	29.0139	0.0888	2	9	negative regulation of osteoblast proliferation
GO:0045869	0.0033	29.0139	0.0888	2	9	negative regulation of single stranded viral RNA replication via double stranded DNA intermediate
GO:0070383	0.0033	29.0139	0.0888	2	9	DNA cytosine deamination
GO:0072048	0.0033	29.0139	0.0888	2	9	renal system pattern specification
GO:0051270	0.0034	2.1328	9.579	19	971	regulation of cellular component movement
GO:0043001	0.0035	10.9332	0.3058	3	31	Golgi to plasma membrane protein transport
GO:0032409	0.0035	3.421	2.4761	8	251	regulation of transporter activity
GO:0071526	0.0038	10.5555	0.3157	3	32	semaphorin-plexin signaling pathway
GO:0043648	0.0038	5.1432	1.0358	5	105	dicarboxylic acid metabolic process
GO:0001756	0.0041	6.6105	0.6511	4	66	somitogenesis
GO:0009972	0.0041	25.3857	0.0987	2	10	cytidine deamination
GO:0046087	0.0041	25.3857	0.0987	2	10	cytidine metabolic process
GO:0035510	0.0041	10.2031	0.3255	3	33	DNA dealkylation
GO:0048870	0.0043	1.8716	15.0936	26	1530	cell motility
GO:0070988	0.0048	6.3043	0.6807	4	69	demethylation
GO:0030307	0.0048	4.0729	1.5587	6	158	positive regulation of cell growth
GO:0034754	0.0049	4.8503	1.095	5	111	cellular hormone metabolic process
GO:0060766	0.005	22.5637	0.1085	2	11	negative regulation of androgen receptor signaling pathway
GO:0007045	0.0056	6.0251	0.7103	4	72	cell-substrate adherens junction assembly
GO:0060429	0.0058	1.9184	12.3412	22	1251	epithelium development
GO:0001867	0.006	20.3061	0.1184	2	12	complement activation, lectin pathway
GO:0016554	0.006	20.3061	0.1184	2	12	cytidine to uridine editing
GO:0046133	0.006	20.3061	0.1184	2	12	pyrimidine ribonucleoside catabolic process
GO:0072520	0.006	20.3061	0.1184	2	12	seminiferous tubule development
GO:0048513	0.0067	1.5794	33.344	47	3380	animal organ development
GO:0032101	0.0068	2.1741	7.3298	15	743	regulation of response to external stimulus
GO:0001838	0.007	4.4295	1.1937	5	121	embryonic epithelial tube formation
GO:0045995	0.007	4.4295	1.1937	5	121	regulation of embryonic development
GO:0010566	0.007	18.459	0.1282	2	13	regulation of ketone biosynthetic process
GO:0002699	0.007	3.7491	1.6869	6	171	positive regulation of immune effector process
GO:0016053	0.0076	2.6059	4.0447	10	410	organic acid biosynthetic process
GO:0045668	0.0076	8.0512	0.4045	3	41	negative regulation of osteoblast differentiation
GO:0090382	0.0076	8.0512	0.4045	3	41	phagosome maturation
GO:0050772	0.0077	5.4604	0.7793	4	79	positive regulation of axonogenesis
GO:1901888	0.0081	5.3882	0.7892	4	80	regulation of cell junction assembly
GO:0000722	0.0081	16.9197	0.1381	2	14	telomere maintenance via recombination
GO:0042446	0.0084	5.3179	0.7991	4	81	hormone biosynthetic process
GO:0001667	0.0085	2.56	4.1137	10	417	ameboidal-type cell migration
GO:0030278	0.0092	3.5327	1.7856	6	181	regulation of ossification
GO:0010959	0.0092	2.6793	3.5317	9	358	regulation of metal ion transport
GO:1904062	0.0094	2.8684	2.9299	8	297	regulation of cation transmembrane transport
GO:0000415	0.0099	Inf	0.0099	1	1	negative regulation of histone H3-K36 methylation
GO:0003147	0.0099	Inf	0.0099	1	1	neural crest cell migration involved in heart formation
GO:0030209	0.0099	Inf	0.0099	1	1	dermatan sulfate catabolic process
GO:0035713	0.0099	Inf	0.0099	1	1	response to nitrogen dioxide
GO:0044345	0.0099	Inf	0.0099	1	1	stromal-epithelial cell signaling involved in prostate gland development
GO:0046901	0.0099	Inf	0.0099	1	1	tetrahydrofolylpolyglutamate biosynthetic process
GO:0048694	0.0099	Inf	0.0099	1	1	positive regulation of collateral sprouting of injured axon
GO:0050928	0.0099	Inf	0.0099	1	1	negative regulation of positive chemotaxis
GO:0060598	0.0099	Inf	0.0099	1	1	dichotomous subdivision of terminal units involved in mammary gland duct morphogenesis
GO:0061713	0.0099	Inf	0.0099	1	1	anterior neural tube closure
GO:0061767	0.0099	Inf	0.0099	1	1	negative regulation of lung blood pressure
GO:0071250	0.0099	Inf	0.0099	1	1	cellular response to nitrite
GO:0071954	0.0099	Inf	0.0099	1	1	chemokine (C-C motif) ligand 11 production
GO:0072168	0.0099	Inf	0.0099	1	1	specification of anterior mesonephric tubule identity
GO:0072169	0.0099	Inf	0.0099	1	1	specification of posterior mesonephric tubule identity
GO:0072184	0.0099	Inf	0.0099	1	1	renal vesicle progenitor cell differentiation
GO:0072259	0.0099	Inf	0.0099	1	1	metanephric interstitial fibroblast development
GO:0090246	0.0099	Inf	0.0099	1	1	convergent extension involved in somitogenesis
GO:0098749	0.0099	Inf	0.0099	1	1	cerebellar neuron development
GO:1900281	0.0099	Inf	0.0099	1	1	positive regulation of CD4-positive, alpha-beta T cell costimulation
GO:1904328	0.0099	Inf	0.0099	1	1	regulation of myofibroblast contraction
GO:1904635	0.0099	Inf	0.0099	1	1	positive regulation of glomerular visceral epithelial cell apoptotic process
GO:1904877	0.0099	Inf	0.0099	1	1	positive regulation of DNA ligase activity
GO:1905580	0.0099	Inf	0.0099	1	1	positive regulation of ERBB3 signaling pathway
GO:1905943	0.0099	Inf	0.0099	1	1	negative regulation of formation of growth cone in injured axon
GO:2000080	0.0099	Inf	0.0099	1	1	negative regulation of canonical Wnt signaling pathway involved in controlling type B pancreatic cell proliferation
GO:2000184	0.0099	Inf	0.0099	1	1	positive regulation of progesterone biosynthetic process
GO:2000572	0.0099	Inf	0.0099	1	1	positive regulation of interleukin-4-dependent isotype switching to IgE isotypes

**Table 6:** GO enrichment analysis showing the significant molecular functions (MF) of the top 500 hypomethylated promoters.

GOMFID	P-value	Odds ratio	ExpCount	Count	Size	Term
GO:0036374	0	105.3038	0.0575	3	6	glutathione hydrolase activity
GO:0047844	0.0019	41.8516	0.0671	2	7	deoxycytidine deaminase activity
GO:0000979	0.0045	9.855	0.3354	3	35	RNA polymerase II core promoter sequence-specific DNA binding
GO:0004126	0.0057	20.9195	0.115	2	12	cytidine deaminase activity
GO:0050681	0.0075	8.0828	0.4025	3	42	androgen receptor binding
GO:0031492	0.0091	7.5041	0.4312	3	45	nucleosomal DNA binding
GO:0003940	0.0096	Inf	0.0096	1	1	L-iduronidase activity
GO:0004326	0.0096	Inf	0.0096	1	1	tetrahydrofolylpolyglutamate synthase activity
GO:0004441	0.0096	Inf	0.0096	1	1	inositol-1,4-bisphosphate 1-phosphatase activity
GO:0008725	0.0096	Inf	0.0096	1	1	DNA-3-methyladenine glycosylase activity
GO:0008829	0.0096	Inf	0.0096	1	1	dCTP deaminase activity
GO:0008841	0.0096	Inf	0.0096	1	1	dihydrofolate synthase activity
GO:0031962	0.0096	Inf	0.0096	1	1	mineralocorticoid receptor binding
GO:0034512	0.0096	Inf	0.0096	1	1	box C/D snoRNA binding
GO:0043916	0.0096	Inf	0.0096	1	1	DNA-7-methylguanine glycosylase activity
GO:0050649	0.0096	Inf	0.0096	1	1	testosterone 6-beta-hydroxylase activity
GO:0052821	0.0096	Inf	0.0096	1	1	DNA-7-methyladenine glycosylase activity
GO:0052822	0.0096	Inf	0.0096	1	1	DNA-3-methylguanine glycosylase activity
GO:0052829	0.0096	Inf	0.0096	1	1	inositol-1,3,4-trisphosphate 1-phosphatase activity
GO:0086038	0.0096	Inf	0.0096	1	1	calcium:sodium antiporter activity involved in regulation of cardiac muscle cell membrane potential
GO:0031625	0.0096	2.8578	2.9417	8	307	ubiquitin protein ligase binding
